# 

**Published:** 1827-10

**Authors:** 


### Contents

OF

### No. 14, Vol. VII. (NEW SERIES) for OCTOBER, 1827.

[*FOURTH NUMBER FOR* 1827.]

Art. I.

DUBLIN HOSPITAL REPORTS.

1. Mr. Cusack's Report on Amputation of Portions of the Lower Jaw, in Steeven's Hospital .......... 289

2. Inquiry into the Value of Mediate Auscultation, by Dr. Stack ...... 297

3. Cases of Excision of Carious Joints. By Philip Crampton, Esq..... 301

4. On Dissection Wounds. By A. Colles, M. D. .......... 306

5. A selection of Cases from the Medical Wards of the Meath Hospital, and Dublin Infirmary. By Drs. Graves and Stokes .......... 309

6. Case of Ununited Fracture of the Tibia successfully treated. By Dr. Browne .......... 315

II.

Commentaries on some of the more Important of the Diseases of Females. By Marshall Hall, M. D. .......... 316

1. Disorders incident to Female Youth.......... 317

2. Disorders of the General Health .......... 319

3. Protracted General Disorder .......... 324

4. Chlorosis .......... 326

5. Anomalous Affections.......... 328

6. Hysteria .......... 329

7. Diseases of Females returned from India.......... 330

8. On Local Inflammatory Diseases.......... 330

9. On Tubercular Affections of the Abdomen.......... 334

10. On Affections of the Uterus and Mamma .......... 335

III.

Dr. Scudamore on Rheumatism, Rheumatic Neuralgia, and Tic Douloureux 337

IV.

Dr. Shute on Medical Science and Practice.......... 358

V.

Dr. Philip on Protracted Cases of Indigestion.......... 362

VI.

Dr. Christison on Poisoning by Arsenic .......... 379

VII.

Mr. Wadd's Mems. Maxims, and Memoirs .......... 393

VIII.

Mr. Guthrie on Gunshot Wounds .......... 398

IX.

### Quarterly Periscope

OF

### PRACTICAL MEDICINE.

PART I.

1. M. Cruveilheir on a Peculiar Disease of the Heart .......... 401

2. Various Cases of Neuralgia cured by Carb. Ferri .......... 403

3. Dr. Whoeler's Experiments on the Passage of Substances in the Urine 404

4. Dr. Renton on Dysentery in the Island of Madeira.......... 407

5. M. Barrut on Gastrodynia.......... 408

6. Ascites Complicated with Pregnancy .......................... 409
7. Case of Fatal Salivation .......................................... 409
8. Professor Mayer on Section of the Par Vagum .................. 410
9. Glossitis treated by Incisions...................................... 411
10. The Proteiform Malady, a Remarkable Case....................... 412
11. Successful Transfusion in Uterine Hæmorrhage.................. 415
12. Ligature of the Common Iliac Artery successful ................ 416
13. Cynanche Laryngea requiring Bronchotomy ....................... 417
14. Dr. Mott's Case of Successful Amputation at the Hip-Joint........... 418
15. Cerebral Agenesis, or Defects of Organization in the Brain ........ 420
16. Dr. Musgrave on the Unmixed Effects of Mercury on the System.... 423
17. Obliteration of the Aorta—Scirrhous Diathesis...................... 426
18. Mr. Bell on Strictures of the Rectum .................................. 427
19. General Subcutaneous Emphysema....................................... 429
20. Interesting Case of Angina Pectoris ..................................... 430
21. On Infiltration of the Labia Pudendi in Parturition................ 431
22. Homœopathy—a German Farce ............................................ 432
23. Drs. Barry and Arnott on the Circulation of the Blood.............. 434
24. Remarkable Case of Perforation of the Bladder ...................... 438
25. Fatal Case of Cancer of the Cardiac Orifice of the Stomach ........ 439
26. On the Omission of Ligature in Amputations.......................... 440
27. Remarkable Case of Congenital Enlargement of the Heart ........ 443
28. Mr. Smith on Collared Cases in Midwifery ............................ 444
29. Case of Supposed Hydro-pericardium cured............................ 444

MEDICINA FORENSICA.

30. Correspondence between the Censors of the College of Physicians and Dr. Harrison—approaching Trial .......................................... 445

PART II. HOSPITAL REPORTS.

1. M. Dupuytren on a peculiar Mode of Treating Urethral Strictures—*(Hôtel Dieu)* .......................................... 449
2. Dr. Fearn on the Re-union of Divided Tendons *(Philadelphia Infirmary)* .......................................... 451
3. On the Bad Consequences which sometimes attend the Reduction of Old Dislocations, with Cases. By M. Flaubert—*(Hôtel Dieu de Rouen)* .......................................... 452
4. Case where the Astragalus was extracted—*(New York Hospital)*.... 457
5. Case of Pneumonia and Gastritis, without any Pain—*(Val de Grace)* 458
6. Permanent Evidence of Successful Vaccination. By Dr. Gregory—*(Small Pox Hospital)*.......................................... 459
7. Case of Remarkable Intermittent Hiccup, attended with peculiar Phenomena. By Dr. Hellis—*(Hôtel Dieu de Rouen)* .............. 459
8. Mr. Travers on Diseased and Wounded Arteries—*(St. Thomas's Hosp.)* 461
9. M. Ollivier on Inflammation of the Spinal Marrow—*(Necker)* ...... 464
10. Hypertrophy of the Heart, Remarkable Case of—*(Evreux Gen. Hosp.)* 465
11. Dr. Bouillaud on Pulmonary Apoplexy—*(Hôp. Cochin)* ............ 466
12. Cases of Purpura Hæmorrhagica, with Dissection—*(Hôp. St. Louis)*.. 468
13. Remarkable Tendency to Phlegmasiæ, with Dissection—*(Strasbourg Hospital)* .......................................... 470
14. Mr. Earle on Strangulated Hernia—*(Bartholomew's)* .............. 472
15. Dr. Ranque on Colica Pictonum—*(Hôtel Dieu d'Orleans)* .......... 474
Dr. Pascal on the same Disease—*(Military Hospital of Compostile)*.. 477
16. Case of Ununited Fracture cured by Pressure—*(St. George's Hospital)* 478
17. Drs. Graves and Stokes on Cutaneous Diseases—*(Meath Hospital)*.. 479
18. Fever—Gastro-Enterite—*(Bartholomew's)*.......................... 481
19. Pneumonia mistaken for Hydrothorax—*(Meath Hospital)* .......... 482
20. Diseases of the Brain and Spinal Marrow—*(Meath Hospital)*........ 483
21. Fracture of the Cranium and Spine—*(St. George's Hospital)*........ 485
22. Remarkable Hepatic Tumour—*(Strasbourg Hospital)*............... 486
——— Encephaloid Disease of the Liver, in Private Practice.. 487

23. On Protracted Lactation—*(Metropolitan Infirmary)* .............. 488
24. Insidious Abdominal Inflammation—*(Meath Hospital)* ............ 489
25. Remarkable Case of Empyema—*(Military Hospital)*.............. 491
26. Scirrhous Pancreas—Cataract—*(Hôtel Dieu)* .................... 493
27. Dr. Hewett on the Pathology of Fever—(*St. George's Hospital)* .... 494
28. On Diabetes Mellitus—*(Strasbourg Hospital)* ...................... 496
29. Cephalalgia—Ague—Tubercles—*(Bicêtre)*........................ 497
30. M. Bally on the Seat of Fever—*(La Pitié)*........................ 498
31. Mr. Lee on the Nutrition of the Fœtus—*(Metropolitan Infirmary)*.... 504

### PART III. ANALECTA.

### AN EXAMINATION OF THE INFLUENCE AND OPERATION OF THE ROYAL COLLEGE OF PHYSICIANS, BY ITS CHARTER AND LAWS, ON THE PHYSICIANS OF THE UNITED KINGDOM.

Charter of Incorporation, Original and Translation ................. 505
Ostensible and bonâ fide Objects of the Charter ........................ 509
Charter enjoins the Association of *all* properly qualified Physicians, as one Body or Commonalty.......................................... 509
Charter makes no distinction between Fellows and Licentiates.......... 509
This distinction inconsistent with the Spirit of the Charter ............ 509
——————— degrades one Class and elevates the other.............. 510
Remarks on the real or supposed Advantages of the Oxford and Cambridge Education ............................................................. 511
Inconsistency of confining the *Classical* part of a Physician's Education to the Universities, leaving the Medical part free........................ 511
Dereliction of the College in not punishing Quacks........................ 512
Dereliction of the College in Licensing certain Physicians ............ 512
Necessity of returning to the Original Charter.............................. 512
Suggestion respecting a Petition to the Legislature........................ 513
Examination of the Charter in a legal point of view....................... 514
Questions resolved by the Lord Chancellor in the reign of King James.. 514
Letter of King CHARLES making the first distinction between Fellows and Licentiates ........................................................... 515
Criticisms on this Letter, and the probable Cause of it................... 516
King Charles's Letter never confirmed by Parliament ................... 516
Remarkable Trial of Dr. Bonham ............................................. 517
Reflexions on this, and on the approaching Trial of Dr. Harrison......... 517
Chief Justice Coke's Opinion respecting the Characters which are to be prosecuted by the College ............................................ 518
Characters admissible into the Commonalty of the College ............ 519
Remarkable Trial of Dr. Groenvelt, for disobedience..................... 520

### CRITICAL ANALYSIS OF SELECT HOSPITAL REPORTS IN THE LANCET.

1. Acupuncture in Sciatica.............................................. 521

2. Cases of Hernia at Bartholomew's ..................................... 521

3. Softening of the Brain " Hospital of Surgery"......................... 522

4-5. Congenital Division of the Palate—Abscess of the Liver............ 522

6-7. Curious Appearance in a Stump—Carcinoma of the Palate........ 523

8. Various Cases of Strangulated Hernia................................ 524

9. Compound Fracture of the Cranium.................................... 525

10. Horny Excrescence—Lithotomy in Old Persons...................... 526

11. Excessive Inflammation of the Prepuce ............................ 526

12. Wound of the Brachial Vein, with Criticisms ...................... 527

13. Wound of the Knee Joint ............................................. 528

14. Chronic Enlargement of the Testicle ................................ 529

15. Chloride of Lime in Phagedæna Gangrenosa......................... 529

16. Prurigo cured by Colchicum ......................................... 529

17. Compound Fracture of the Lower Jaw ............................... 529

18. Affections of the Knee Joint—Wonderful Discoveries ! ............ 530

19. Ulcer of the Tongue .................................................. 531

20. Sloughing Chancre .................................... 531
21. Fatal Wound of the Larynx .................................... 531
22. Cases of Lithotomy (original and selected) .................................... 532
23. Aneurism of the Subclavian and Innominata .................................... 533
24. Aneurism of the Subclavian with Criticisms .................................... 534
25. Death from Syncope, after Incisions in the Arm ! .................................... 536

PRIZE HOSPITAL REPORT, FROM ST. BARTHOLOMEW'S HOSPITAL.

(Prize adjudged to Mr. FEREDAY, Dresser.)

No. III.

1. Three Cases of Fatal Phlebitis, with the Dissections .................................... 537
2. Case of Traumatic Tetanus, with the Dissection .................................... 543
3. Excision of a very large Polypus Uteri .................................... 545
4. Three Cases of Fractured Ribs, with Emphysema .................................... 545
5. Consequences of Protracted Strangulation in Hernia .................................... 549
6. On the Effects of Local Sedatives in Phagedænic Ulcer, with Cases .. 551
   Proto-Nitrate of Mercury in Phagedænic Ulcer .................................... 553
   Remarks on Sloughing Phagedæna .................................... 554
7. Case of Ulceration of the Larynx, with Dissection .................................... 554
8. Case of Syphilitic Affections in Mother and Infant .................................... 555
9. On Erysipelas Phlegmonodes, treated by Incisions .................................... 556
10. On Erysipelas Œdematodes, with Dissection .................................... 558
11. On the Treatment of Syphilitic Iritis, with Cases .................................... 559
12. Puncture of the Bladder above the Pubes .................................... 561
13. Aneurism of the Femoral Artery .................................... 562
14. Cases of Disease of the Synovial Membrane of the Knee .................................... 563
15. Osteo-Sarcomatous Tumour of the Lower Jaw .................................... 565
16. Cancer Scroti .................................... 566
17. Carcinoma of the Hand, with Operation .................................... 567

PRIZE HOSPITAL REPORT, FROM THE CHELTENHAM CASUALTY HOSPITAL,

(Prize adjudged to MR. TURNER.)

No. IV.

1. Cases of Chorea cured by Carbonate of Iron .................................... 569
2. Case of Cephalalgia cured by Iodine .................................... 570
3. Cases of Gonorrhœa with Irritable Sores .................................... 571
4. Aneurism of the Aorta, bursting into the Chest .................................... 572
5. Injury of the Head .................................... 573
6. Injury of a Nerve in Bleeding .................................... 573
7. Fractured Femur with Hæmoptysis .................................... 574
8. Cataract, Operation for .................................... 574
9. Amputation of the Thigh through the Trochanter .................................... 575
   Amputation of the Leg .................................... 575
10. Osteo-Sarcoma of three Fingers .................................... 576
11. Strangulated Femoral Hernia .................................... 576
12. New Method of Closing the Mouths of Jars containing Wet Anatomical Preparations .................................... 577
Bibliographical Record .................................... 578

INTELLIGENCE, &c.

Obituary—Mr. Shaw .................................... 581
Artificial Anatomical Preparations .................................... 582
Dr. Hopkins' Obstetric Machine .................................... 582
Dublin Hospital Reports .................................... 582
The Contented Helot, or Man in Mask .................................... 583
Anatomical Persecution .................................... 583
Omission of Cancel .................................... 583
Hospital Reports .................................... 584
Chlorides of Lime and Soda .................................... 585

